# New Genus *Fibralongavirus* in Siphoviridae Phages of *Staphylococcus pseudintermedius*

**DOI:** 10.3390/v11121143

**Published:** 2019-12-10

**Authors:** Michal Zeman, Pavol Bárdy, Veronika Vrbovská, Pavel Roudnický, Zbyněk Zdráhal, Vladislava Růžičková, Jiří Doškař, Roman Pantůček

**Affiliations:** 1 Department of Experimental Biology, Faculty of Science, Masaryk University, Kotlářská 2, 611 37 Brno, Czech Republic; 2 Central European Institute of Technology, Masaryk University, Kamenice 5, 625 00 Brno, Czech Republic; 3 National Centre for Biomolecular Research, Faculty of Science, Masaryk University, Kamenice 5, 625 00 Brno, Czech Republic

**Keywords:** bacteriophages, *Staphylococcus pseudintermedius*, viral taxonomy, Siphoviridae, comparative genomics

## Abstract

Bacteriophages of the significant veterinary pathogen *Staphylococcus pseudintermedius* are rarely described morphologically and genomically in detail, and mostly include phages of the Siphoviridae family. There is currently no taxonomical classification for phages of this bacterial species. Here we describe a new phage designated vB_SpsS_QT1, which is related to phage 2638A originally described as a *Staphylococcus aureus* phage. Propagating strain *S. aureus* 2854 of the latter was reclassified by *rpoB* gene sequencing as *S. pseudintermedius* 2854 in this work. Both phages have a narrow but different host range determined on 54 strains. Morphologically, both of them belong to the family Siphoviridae, share the B1 morphotype, and differ from other staphylococcal phage genera by a single long fibre at the terminus of the tail. The complete genome of phage vB_SpsS_QT1 was sequenced with the IonTorrent platform and expertly annotated. Its linear genome with cohesive ends is 43,029 bp long and encodes 60 predicted genes with the typical modular structure of staphylococcal siphophages. A global alignment found the genomes of vB_SpsS_QT1 and 2638A to share 84% nucleotide identity, but they have no significant similarity of nucleotide sequences with other phage genomes available in public databases. Based on the morphological, phylogenetic, and genomic analyses, a novel genus *Fibralongavirus* in the family Siphoviridae is described with phage species vB_SpsS_QT1 and 2638A.

## 1. Introduction

*Staphylococcus pseudintermedius* is a member of the Staphylococcus intermedius group (SIG), which is mostly associated with veterinary pathogens. Staphylococcal isolates from dogs previously identified as *Staphylococcus intermedius* belong almost exclusively to the species *S. pseudintermedius* [1]. Differentiation between species was made possible by modern molecular diagnostic methods based on polymerase chain reaction-restriction fragment length polymorphism [2], repetitive element sequence-based PCR fingerprinting [3] and multi-locus sequence analysis [4]. *S. pseudintermedius* is recognised as an important causative agent of pyoderma and dermatitis in canines, felines, and other animals [5]. Its host range is not restricted to animals, but also has the potential to cause zoonotic infections [6] or endocarditis in humans [7]. Similar to *Staphylococcus aureus*, there is a threat of antimicrobial resistance and the emergence of methicillin-resistant *S. pseudintermedius* (MRSP) strains [8]. The importance of this problem is underlined by the “Reflection Paper on Methicillin-Resistant *S. pseudintermedius*” from the European Medicines Agency [9]. The European Medicines Agency even suggests phage therapy as a possible way to control MRSP.

Most of the phage studies were performed before their subdivision into SIG members and focused on the phage typing of *Staphylococcus intermedius* [10,11,12]. There are only rare reports of staphylococcal Myoviridae phages that act against *S. pseudintermedius* [13]. A group of Siphoviridae phages active against *S. pseudintermedius* was characterized with the aim of therapeutically using them against MRSP [14]. In this paper, we report the description of the new morphologically distinctive genus, *Fibralongavirus,* and a new representative of this genus, the phage vB_SpsS_QT1 (abbreviated as QT1). This proposition was already accepted by the International Committee on the Taxonomy of Viruses (ICTV) but has not been ratified yet.

## 2. Materials and Methods

### 2.1. Bacterial Strains and Bacteriophages

Phage 2638A (= HER 283) and its propagation strain *S. pseudintermedius* 2854 (= HER 1283) were purchased from the Félix d’Hérelle Reference Center for Bacterial Viruses (Quebec City, QC, Canada). Fifty-two *S. pseudintermedius* and two *S. intermedius* strains characterized previously [15,16] and reference strains of SIG group obtained from the Czech Collection of Microorganisms (Masaryk University, Brno, Czech Republic) were used to determine the host range (Table S1). Phage QT1 and its propagating strain *S. pseudintermedius* 625 were deposited in the Czech Collection of Microorganisms under numbers CCM 9019 (= QT1) and CCM 9018 (= strain 625).

### 2.2. Growth Properties

Phage host range was determined by spotting serial dilutions on double-layer agar plates with tryptone soya agar (TSA) bottom agar (CM0131, Oxoid, Basingstoke, United Kingdom) and 0.6% (*w*/*v*) top Agar No. 1 (LP0011, Oxoid) with the addition of CaCl_2_ to a concentration of 2 mM. The growth properties and adsorption efficiency of phages were determined in tryptone soya broth (TSB) (CM0129, Oxoid), with 2 mM CaCl_2_ as described previously [17] with minor modification for staphylococci [18]. The ability to grow on different media was further tested on meat peptone agar (MPA) and double-concentrated yeast extract-tryptone (2× YT) agar composed of 1.6% (*w*/*v*) tryptone (LP0042, Oxoid), 1.0% (*w*/*v*) yeast extract (LP0021, Oxoid), 0.5% (*w*/*v*) NaCl, and 1.5% (*w*/*v*) agar (LP0013, Oxoid), pH 7.0. A one-step growth curve and phage burst size were determined by the procedure described previously [17]. Free plasmacoagulase and clumping factor tests were performed as described previously [19,20].

### 2.3. Phage Propagation and Purification

Phages were propagated using a TSB liquid broth incubation technique with the addition of CaCl_2_ to a final concentration of 2 mM. The phage lysate was centrifuged twice for 30 min at 3100 g and filtered through a 0.45 µm polyethersulfone filter (Techno Plastic Products, Trasadingen, Switzerland). Phage particles were purified in a CsCl density gradient as described previously [21].

### 2.4. Electron and Cryo-Electron Microscopy

Purified phage was diluted to the density A_280 nm_ = 1.0 and incubated with 1 µg mL^−1^ DNase I (Sigma-Aldrich, St. Louis, MO, USA) for 30 min at room temperature. For the negative stain, 4 µL of sample was applied onto glow-discharged carbon-coated 400 mesh copper Quantifoil^®^ grids (Quantifoil Micro Tools, Großlöbichau, Germany) for 1 min. The grids were then washed twice on a drop of deionized water and stained with 2% (*w*/*v*) uranyl acetate. For cryo-EM, 3.9 µL of sample was applied onto glow-discharged R2/1 300 mesh holey carbon Quantifoil^®^ grids, blotted and plunge-frozen in liquid ethane using a Vitrobot Mark IV (Thermo Fisher Scientific, Waltham, MA, USA). The samples were analyzed with a Tecnai F20 TEM (Thermo Fisher Scientific) operated at 200 kV.

### 2.5. DNA Extraction from the Phage Particles

DNase I (Sigma-Aldrich) and RNase A (Sigma-Aldrich) treatment was performed to remove any exogenous host genomic DNA and RNA from purified phage particles as previously described [21]. DNA from the viral particles for sequencing was purified by phenol-chloroform extraction [21]. The concentration and purity of phage DNA was determined using a NanoDrop spectrophotometer (Thermo Fisher Scientific).

### 2.6. Partial rpoB Gene Sequencing and Phylogenetic Analysis

Partial *rpoB* gene amplification was performed as described previously [22]. PCR amplicons were sequenced by Sanger sequencing with the primers 1418F and 1876R in the Eurofins Genomics sequencing facility (Ebersberg, Germany).

### 2.7. Genome Sequencing and Bioinformatic Analyses

Phage genome and bacterial whole-genome shotgun (WGS) sequencing was performed using an Ion Torrent™ Personal Genome Machine (Ion PGM™, Thermo Fisher Scientific). Bacterial genomic DNA was extracted using a High Pure PCR Template Preparation Kit (Roche Diagnostics, Mannheim, Germany) with 5 mg mL^−1^ lysostaphin (Ambi Products, Lawrence, NY, USA) added to the suspension buffer. The purified genomic DNA was used for preparing a 400-bp sequencing library with an Ion Plus Fragment Library Kit (Thermo Fisher Scientific). The sample was loaded onto a 316v2 chip and sequenced using an Ion PGM Hi-Q sequencing kit (Thermo Fisher Scientific). The physical ends of the phage genome were resolved by the primer walking strategy using ligated QT1 DNA concatemers with T4 DNA ligase (New England Biolabs, Ipswich, MA, USA) as the template for a 597 bp fragment amplified with the primers QT1end_FW (AAAGCACGGCAGATTTGAAC) and QT1end_RV (CGTCACGTATTTTGGGGTCT) and sequenced in Eurofins Genomics (Ebersberg, Germany).

The quality of sequencing reads was analysed by FastQC v0.11.8 [23]. De novo assembly and error correction of raw reads was performed by SPAdes v3.12 [24] with all k-mers 21-127 and follow-up mismatch correction. Genome assembly evaluation was performed by QUAST v5.0.1 [25].

Sequences were manipulated and inspected in the cross-platform bioinformatics software Ugene v1.31.1 [26]. Pairwise global alignments of protein sequences were computed using Emboss stretcher [27]. The primal analysis of sequences was combined from open reading frames (ORFs) prediction using GeneMark.hmm [28] and automatic annotation by RAST v2.0 (genetic code 11, RASTtk annotation scheme) [29]. Gene content was further examined by BLASTp searches in protein sequence databases, pVOGs [30], CD-Search [31], and InterPro v.59 [32]. Then, tRNAscan-SE [33], RNAmmer v.1.2 [34], and hmmsearch v.3.0 [35] were used to analyse functional RNAs. Virulence genes were detected with Abricate v0.8.10 [36] using the databases CARD [37], Resfinder [38], NCBI [39], ARG-ANNOT [40] and VFDB [41]. The protein secondary structure was predicted with Jpred4 [42]. HeliQuest [43] and I-TASSER [44] were used for further structural predictions. Multiple sequence alignments were visualized using EasyFig v.2.1 [45].

Phylogenetic analyses of the β subunit of bacterial RNA polymerase, large subunit of terminase, and major capsid protein were done on the NGPhylogeny.fr server using “PhyML+SMS/OneClick” mode [46] and visualized in FigTree v1.4.3 [47]. The whole-genome comparison of phage genomes was calculated using Gegenees v3.0beta [48] with the blastn algorithm on fragment size = 50 and step size = 25 and genomes were sorted by an internal automatic algorithm.

### 2.8. Pulsed-Field Gel Electrophoresis.

One µg of phage DNA was loaded onto 1.5% (*w*/*v*) agarose gel (Serva, Heidelberg, Germany) and separated with Cheff Mapper (Bio-Rad, Hercules, CA, USA) to detect concatemerised cohesive ends. A constant voltage of 5 V cm^−1^ and pulse times of 2–20 s with linear ramping were applied for 20 h. Lambda DNA concatemers (Sigma-Aldrich) were used as molecular weight marker.

### 2.9. SDS-PAGE of Structural Proteins

Vertical one-dimensional electrophoresis (1-DE) was performed in discontinuous 12% acrylamide SDS-PAGE gel using a Protean II xi Cell (Bio-Rad). Blue Prestained Protein Standard, Broad Range (11–190 kDa) (New England Biolabs) was applied as the molecular weight marker. Proteins were stained with a homemade BlueSilver (100 g ammonium sulphate, 117.6 mL 85% (*w*/*w*) H_3_PO_4_, 200 mL methanol, and 1.2 g Coomassie blue G 250 filled with distilled water to 1 L) [49].

### 2.10. Filter-Aided Sample Preparation for Mass Spectrometry and LC-MS/MS Analysis

The protein solution was processed by the filter-aided sample preparation (FASP) method [50], using trypsin (1 μL, 0.5 μg μL^−1^, sequencing grade, Promega, Madison, WI, USA) for digestion (incubated for 18 h at 37 °C, enzyme: protein ratio 1:50). The resulting peptides were directly extracted into LC-MS vials with 2.5% formic acid in 50% acetonitrile (ACN) and 100% ACN with the addition of polyethylene glycol (20,000; final concentration 0.001%) [51] and concentrated in a SpeedVac concentrator (Thermo Fisher Scientific) prior to LC-MS analyses.

Liquid chromatography–mass spectrometry (LC-MS/MS) analysis of the peptide mixture was done using an RSLCnano system (Thermo Fisher Scientific) online connected to an Impact II Ultra-High Resolution Qq-Time-Of-Flight mass spectrometer (Bruker, Billerica, MA, USA). Peptides were concentrated in a trapping column (100 μm × 30 mm; 3.5-μm X-Bridge BEH 130 C18 sorbent, Waters, Milford, MA, USA) and separated in an Acclaim Pepmap100 C18 analytical column (3 µm particles, 75 μm × 500 mm; Thermo Fisher Scientific). MS data were acquired in a data-dependent strategy with a 3-s cycle time. The mass range was set to 150–2200 m/z and precursors were selected from 300–2000 m/z.

The obtained MS/MS spectra were analyzed in Proteome Discoverer software v1.4 (Thermo Fisher Scientific) using In-house Mascot v2.5.1 (Matrix Science, London, UK) search engine. Mascot MS/MS ion searches were done separately against the provided databases—the QT1 proteome (60 protein sequences), *S. pseudintermedius* 625 prophages (182 protein sequences) and the *Staphylococcus* spp. database UniRef100_Staphylococcus (339,865 protein sequences). The cRAP contaminant database (downloaded from http://www.thegpm.org/crap/) was searched in advance to exclude contaminant spectra prior to the main database search. The mass tolerances for peptides and MS/MS fragments were 25 ppm and 0.05 Da, respectively. The oxidation of methionine, carbamidomethylation (C), and deamidation (N, Q) were set as variable modifications, and two enzyme miscleavages were allowed for all searches. Rank 1 peptides with a minimum length of 6 amino acids and Mascot expectation value < 0.01 were used for protein list generation. Proteins identified based on at least two peptides are reported.

### 2.11. Nucleotide Sequence Accession Numbers

The complete genome of the *S. pseudintermedius* phage vB_SpsS_QT1 was deposited in the GenBank database under the accession number MK450538. A partial sequence of the *rpoB* gene from *S. pseudintermedius* HER 1283 was deposited in GenBank with the accession number MN564940. The data from the WGS of the bacterial propagating strain *S. pseudintermedius* 625 were recorded in the GenBank WGS project under the accession number WJOJ00000000. The sequence of p222-like_Sps625 plasmid was deposited in GenBank under the accession number WJOJ01000027.

## 3. Results

### 3.1. Growth Characteristics of Phage QT1

Phage QT1 was isolated from rare plaques found in a culture of *S. pseudintermedius* strain 625 on an agar plate. The phage-containing material was transferred to TSB and further propagated on strain 625. Phage QT1 was able to grow on TSA, MPA and 2× YT agar, and formed clear plaques with a diameter of 1.15 ± 0.077 mm (*n* = 30) on TSA with soft agar. Phage QT1 was able to propagate both in liquid broth and on the agar plates using the double-layer agar technique. About 5%–10% of plaques were turbid, but we were unable to derive lysogens of phage QT1 on its propagating strain. The adsorption of the phage was determined on three strains (Figure S1), namely propagating strain *S. pseudintermedius* 625, phage-resistant *S. pseudintermedius* 408, and *S. pseudintermedius* 73, which exhibited an inhibition of growth with no plaques at higher dilutions. The adsorption constant for system phage QT1 and propagating strain *S. pseudintermedius* 625 was calculated as k = 2.98 × 10^−9^ mL min^−1^. One minute after the inoculation of the host bacteria, about 20% of the phage particles remained unadsorbed in supernatants in all strains. After 10 min 97%, 91% and 77% of phages were adsorbed on *S. pseudintermedius* strains 625, 73 and 408, respectively (Figure S1). The latent period until the release of new progeny was 55 min. The burst size determined on the propagating strain was 76 phages per cell.

### 3.2. Virion Morphology of Phages QT1 and 2638A

**Figure 1:**
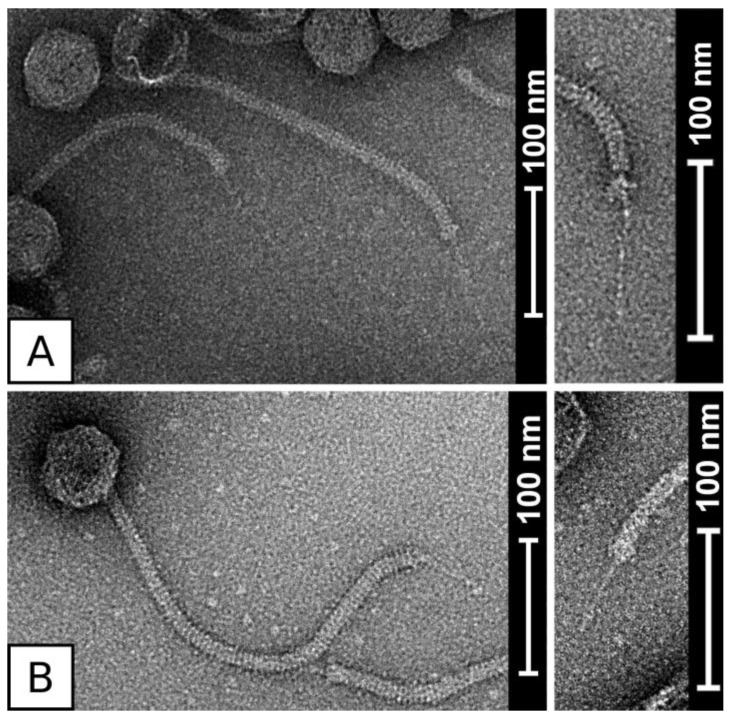
Micrographs of negatively stained phages QT1 (**A**) and 2638A (**B**), with detail on the tail fibre on the right side. The phage suspension was treated with DNase I.

Micrographs from negative stain transmission electron microscopy show that phage QT1 belongs to the family Siphoviridae and has the B1 morphotype (Figure 1). Due to artefacts caused by staining, the dimensions of virion were determined from native cryo-EM samples (Figure S2). The phage head has a diameter of 61 ± 2.3 nm (*n* = 10), its long non-contractile tail has a length of 320 ± 7.4 nm (*n* = 10) and ends with a single tail fibre from the bottom of the tail with a length of about 70 ± 2.0 nm (*n* = 9). The tail has a width of about 10 nm. The length of the whole virion is about 445 nm. The morphologically most similar phage to QT1 is phage 2638A. The tail fibre of phage 2638A is shorter than that of QT1, with a length of 53 ± 3.1 nm (*n* = 11) (Figure 1).

### 3.3. Reclassification of the Propagating Strain Staphylococcus aureus 2854 for Phage 2638A as Staphylococcus pseudintermedius and Phage Host Range

**Figure 2:**
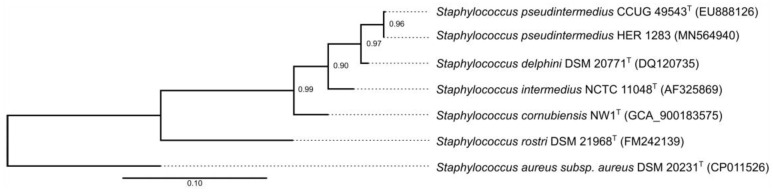
Maximum-likelihood phylogenetic tree based on partial *rpoB* gene sequences of type species of Staphylococcus intermedius group and propagating strain for phage 2638A (*S. pseudintermedius* HER 1283). *S. aureus* was used as a reference. GenBank accession numbers to partial *rpoB* gene sequences are in brackets except for *S. cornubiensis,* where the GenBank assembly accession number is indicated. Shimodaira-Hasegawa-like support values from approximate likelihood-ratio test are indicated at the branching points. The scale represents nucleic acid substitutions per site.

Phage 2638A was originally described as a phage of *Staphylococcus aureus* [52,53], however phylogenetic analysis based on partial *rpoB* sequencing, which was previously shown to be suitable for the identification of staphylococcal species [22], classified its propagating strain as *S. pseudintermedius* (Figure 2).

The host range of both phages QT1 and 2638A was determined on a set of 54 SIG strains (Table S1). Only 13 strains (24%) exhibited sensitivity to QT1 and/or 2638A. About 27% of insensitive strains exhibited inhibition of growth, when undiluted or 10× diluted phage lysate was used, with no plaques at higher dilutions.

### 3.4. Phage Structural Proteins

**Figure 3:**
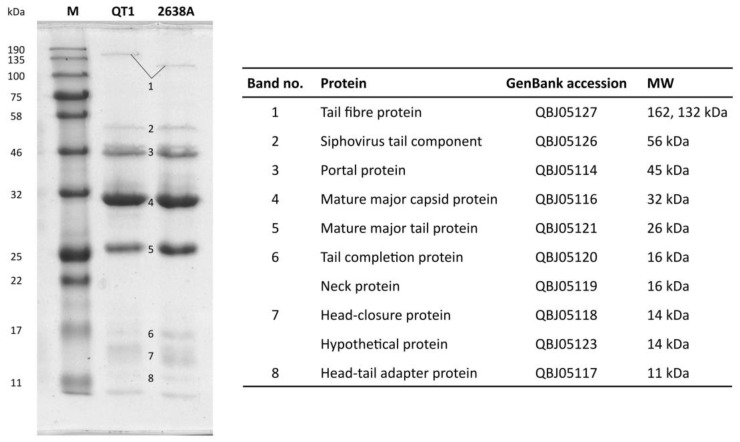
Coomassie brilliant blue-stained 1D SDS-PAGE gel with QT1 and 2638A virion structural proteins. Proteins were extracted from phage particles purified in a CsCl density gradient. All described proteins were confirmed by liquid chromatography-mass spectrometry for phage QT1. GenBank accession numbers for phage QT1 proteins are shown. M, Blue Prestained Protein Standard-Broad Range (New England Biolabs); MW, molecular weight.

Structural proteins of both phages have similar patterns visible on 1D SDS-PAGE, with the only difference being in the putative tail fibre protein (Figure 3). A significant difference is apparent in the molecular weight of the tail fibre, estimated to be 162 kDa and 132 kDa for phages QT1 and 2638A, respectively.

### 3.5. QT1 Genome Description

**Figure 4:**
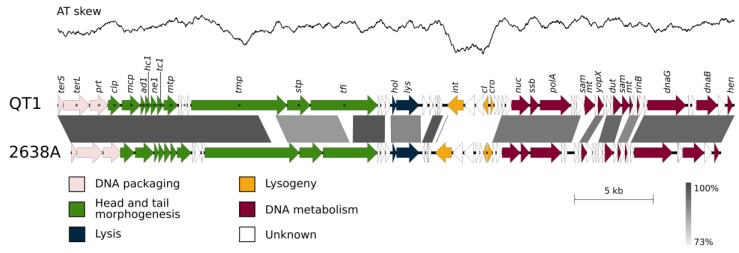
Genome comparison of *S. pseudintermedius* phages QT1 (GenBank accession number MK450538) and 2638A (GenBank accession number NC_007051). The genomes were aligned using the blastn algorithm and similar regions with more than 73% identity are indicated. The position and orientation of predicted genes is represented by arrows. Genome modules are colour coded according to the legend. AT skew for phage QT1 was drawn over a 1000 bp-long window with a step every 1 nt. * denotes proteins from the DNA packaging and head and tail morphogenesis modules of QT1 detected by mass spectrometry.

The genome of phage QT1 consists of dsDNA and has a length of 43,029 bp and G+C content of 36.9%. The physical ends of its genome are 10-nt-long 5′ end overhangs (CGGCGCTTGG). These cohesive ends allow the formation of concatemers, confirmed by pulsed-field gel electrophoresis (Figure S3). Sixty genes were predicted in the QT1 genome, and no genes for tRNA were found. Its genes are organized into modules typical for staphylococcal Siphoviridae phages (Figure 4). Phages QT1 and 2638A [52] share 84% global nucleotide identity and 7% gaps in a 43,585-nt space (Figure 4). According to CD-HIT and a 40% aa identity threshold, forty-one proteins (68%) are similar between phage QT1 and 2638A. The DNA sequence of the packaging module has 99% identity to that of phage 2638A. Both phages QT1 and 2638A belong to the Type1 (Cluster 2) category of Siphoviridae according to a Virfam [54] analysis of the neck module and part of the head and tail proteins. The module for the morphogenesis of the head and tail is identical except for the tail length tape-measure protein (Tmp). Tmp proteins of QT1 and 2638A have a different COG5412 super family domain in the distinctive protein region. The Tmp of QT1 contains the same type of peptidase M23 domain that is present in QT1 endolysin (29.6% aa identity, 40.8% similarity) and might act as a tail hydrolase. The tail appendices (baseplate) module also shares high identity in both phages, except for the tail fibre protein (Tfi). The tail fibre protein consists of an N-terminal conserved domain that is approximately 300 amino-acids long and there is a 90-aa-long intra-molecular chaperone auto-processing domain at the C terminus of the protein [55]. The rest of the protein does not have any predicted (known) domains and contributes to the majority of the overall protein length. The tail fibre protein in phage 2638A is shorter than the tail fibre in QT1 because of a deletion in the middle of the gene corresponding to 265 aa. The whole structural module shares a high degree of nucleotide identity (88.1%) except for the last gene encoding a DUF2951 domain-containing protein (GenBank accession QBJ05130) adjacent to the holin gene. This protein is just distantly related to its homologue in 2638A (39% identity, 53% similarity).

In the lytic module, endolysins of both phages share a high degree of similarity (96% aa identity, 97% similarity). The region between the phage endolysin and integrase genes that frequently harbours virulence factors in *S. aureus* temperate phages contains a 650-bp long region with 3 hypothetical genes that share 97% nucleotide identity to phage 2638A. The rest of the region is not conserved. 

An integration and lysogeny regulation module encoding a tyrosine-based XerC integrase is completely different in the QT1 and 2638A phages. The region transcribed from the negative strand is visible in the AT-skew plot (Figure 4) as a significant drop in the AT deviation (A−T)/(A+T) value compared to the rest of the genome (window 1000 bp, with 1000 steps per window).

Four genes are different in the DNA metabolism module when comparing the genome of phage QT1 to the genome of phage 2638A. Two of them are hypothetical, but the other two in the QT1 genome were predicted to be SAM methyltransferase genes. However, the DNA of phage QT1 was digested with two methylation-sensitive restriction enzymes, MboI and AvaII (Dam and Dcm sensitive restriction).

Most of the proteins from the packaging module and the head-tail morphogenesis module were detected in virions using LC-MS analyses (Table S2). No small subunit of terminase was observed, and one hypothetical protein (GenBank QBJ05123) encoded by a gene located between the major tail protein and the tail length tape measure protein was observed. The major tail protein is probably proteolytically cleaved and its C-terminal bacterial Ig-like domain is missing in mature virions, revealed as the lack of peptide coverage in MS data. Due to the high sensitivity of mass spectrometry, some proteins from other modules were detected at low quantities even after purification in a CsCl density gradient (Table S2).

### 3.6. Taxonomical Classification and Proposal of New Genus Fibralongavirus

**Figure 5:**
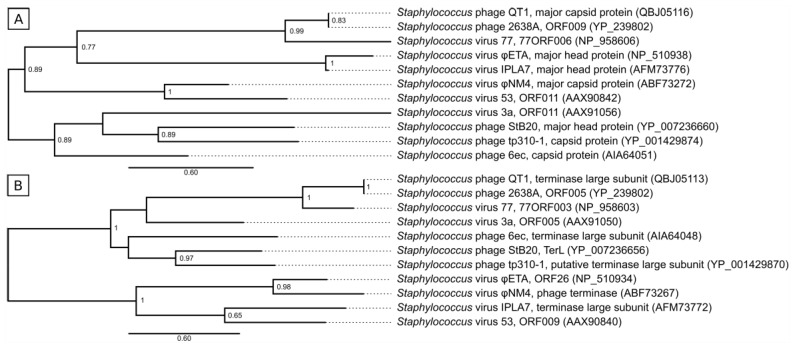
Maximum-likelihood phylogenetic tree based on amino acid sequences of major capsid proteins (**A**) and large subunits of terminase (**B**) of phages QT1 and 2638A and representatives of other *Staphylococcus* phage genera. Phage names were adopted from NCBI, GenBank accession numbers are in brackets. Shimodaira-Hasegawa-like support values from approximate likelihood-ratio test are indicated at the branching points. Scales represent amino acid residue substitution per site.

Phylogenetic analysis of the major capsid protein (Mcp) and large subunit of terminase (TerL) confirmed that phages QT1 and 2638A form a distinct group from members of known staphylococcal phage genera (Figure 5). The closest relative to QT1 and 2638A of other genera is *Staphylococcus aureus* phage 77, a representative of the *Biseptimavirus* genus.

**Figure 6:**
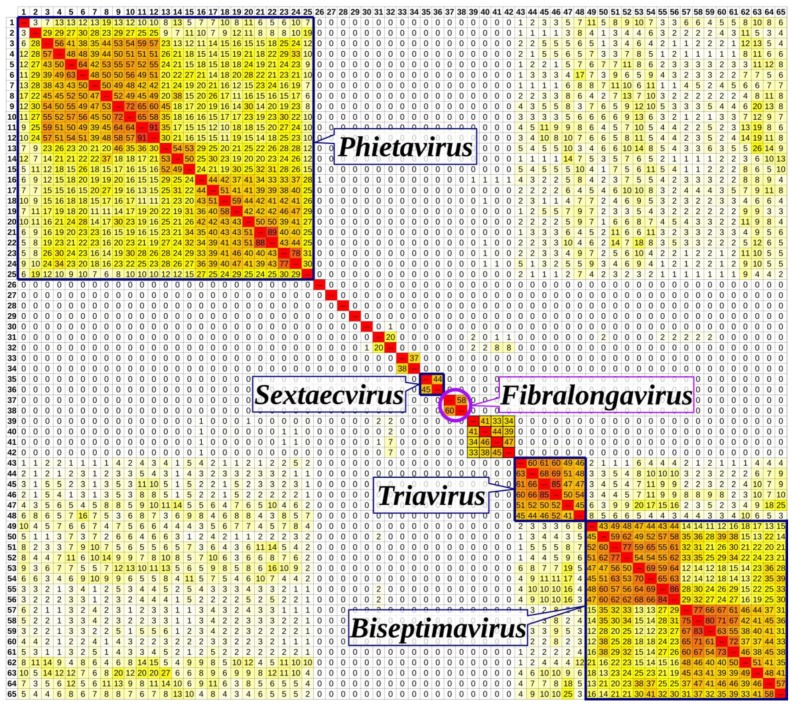
Heat map showing the percent nucleotide similarity of bacteriophage genomes classified by ICTV. Clustering to accepted phage genera according to fragmented nucleotide sequence alignment is indicated in text boxes. Phage names and GenBank accession numbers of used phages labelled as in NCBI are as follows: 1—187, AY954950; 2—φSauS-IPLA88, EU861004; 3—85, AY954953; 4—φMR25, AB370205; 5—69, AY954951; 6—φ11, AF424781; 7—SAP-26, GU477322; 8—φETA2, AP008953; 9—φNM1, NC_008583; 10—φNM2, DQ530360; 11—53, AY954952; 12—80α, DQ517338; 13—φNM4, DQ530362; 14—φETA3, AP008954; 15—96, AY954960; 16—71, AY954962; 17—φETA, AP001553; 18—55, AY954963; 19—φMR11, AB370268; 20—29, AY954964; 21—52A, AY954965; 22—80, DQ908929; 23—92, AY954967; 24—88, AY954966; 25—X2, AY954968; 26—EW, AY954959; 27—φRS7, NC_022914; 28—SpaA1, NC_018277; 29—37, AY954958; 30—IME-SA4, NC_029025; 31—StB20-like, NC_028821; 32—StB20, NC_019915; 33—φ575, KY389063; 34—φ879, KY389064; 35—6ec, KJ804259; 36—vB_SepS_SEP9, KF929199; **37—vB_SpsS_QT1, MK450538**; 38—2638A, NC_007051; 39—vB_SepiS-φIPLA5, JN192400; 40—CNPH82, DQ831957; 41—vB_SepiS-φIPLA7 JN192401; 42—PH15 DQ834250; 43—vB_SauS-φIPLA35 EU861005; 44—3A AY954956; 45—47, AY954957; 46—φ12, AF424782; 47—φSLT, AB045978; 48—42E, AY954955; 49—φPV83, NC_002486; 50—JS01 NC_021773; 51—tp310-3, NC_009763; 52—φ13, AF424783; 53—φPVL108, AB243556; 54—φPVL-CN125, NC_012784; 55—PVL, NC_002321; 56—tp310-1, NC_009761; 57—23MRA, NC_028775; 58—φNM3, NC_008617; 59—StauST398-4, NC_023499; 60—φN315, NC_004740; 61—φBU01, NC_026016; 62—P954, NC_013195; 63—77, AY508486; 64—φSa119, NC_025460; 65—φ5967PVL, NC_019921.

Genomes of staphylococcal siphophages classified by ICTV and phages QT1 and 2638A were compared in Gegenees fragmented aligner (Figure 6). The phages formed clusters that agreed with their classification to viral genera, although some sub-clustering inside known genera was visible. Phages QT1 and 2638A formed a cluster with an overall similarity of 58%–60%, which was separated from other already described genera. On the other hand, they shared less than 1% overall similarity with members of known phage genera. When the phage QT1 genome was compared to all *S. pseudintermedius* Siphoviridae phages and prophages that are publicly available in NCBI databases, prophage φSP119-1 (GenBank accession MK075004) [56] is clustered together with phages QT1 and 2638A (Figure S4). The prophage φSP119-1 genome contains a gene for a tail fibre protein with an expected length of 64 nm calculated from protein sequence data. The integrase of prophage φSP119-1 is only one amino acid different (K241R) from QT1′s integrase, and the prophage is integrated into the tRNA^Ser^ gene. 

All the results support the proposal of a new genus in the family Siphoviridae, for which we suggest the name *Fibralongavirus* with regard to the long terminal tail fibre. Phages QT1 (vB_SpsS_QT1) and 2638A (vB_SpsS_2638A) represent two phage species with 95% genome sequence identity as the criterion for demarcation of the species in this new genus. The major differences between the two species are in the integration module and minor ones in the DNA metabolism module. We propose phage 2638A as the type species for the genus *Fibralongavirus*.

### 3.7. Genome Description of Propagating Strain for Phage QT1

The whole-genome sequencing of propagating strain *S. pseudintermedius* 625 confirmed that phage QT1 was not induced from this strain and was probably free in the mixture with stock culture. However, two prophages other than QT1 are present in the genome of *S. pseudintermedius* 625. The genome of *S. pseudintermedius* strain 625 contains several virulence genes (Table S3). Strain 625 harbours a p222-like plasmid designated as p222-like_Sps625, which is 346 bp longer than p222 and similarly encodes the inducible bacteriocin BacSp222, which is cytotoxic for both gram-positive bacteria and mammalian cells [57]. Six contigs no. 25, 30, 31, 38, 39 and 47 are aligned to a pRE25-like element, described as a mobile genetic element that confers resistance to multiple antibiotics in *S. pseudintermedius* and originating from *Enterococcus* spp. [58].

## 4. Discussion

Many *S. pseudintermedius* isolates predating the description of SIG members are misclassified as *S. aureus* due to the positive plasma coagulase reaction. Also, in propagation strains for *Fibralongavirus* phages, the test for the activity of a clumping factor was positive and the plasmacoagulase test was positive but delayed. Therefore, molecular techniques or MALDI-TOF MS are crucial for the correct differentiation between *S. aureus* and *S. pseudintermedius* [2]. Here, we document a similar case for the host strain HER1283 for phage 2638A, which was described as *S. aureus* prior to the description of *S. pseudintermedius* species, and based on the *rpoB* gene sequencing [59], we confirmed that phage 2638A isolated in the 1980s is a *S. pseudintermedius* phage (Figure 2). Recent advances in diagnostic techniques enable the correct classification of propagating strains, and there is a good chance that hosts of the original phage isolates would belong to different species than those formerly reported.

The host range of the two members of *Fibralongavirus* is different, but narrow (Table S1). Their adsorption kinetics (Figure S1) show that the phages also adsorb onto a resistant strain, suggesting that bacterial resistance is not caused by the absence of a receptor. CRISPR spacers targeting both 2638A and QT1 were found in published *S. pseudintermedius* genomes SP079 and ED99 [60,61], which may be the reason for bacterial resistance to the phages. The CRISPR spacer is identical in both strains and targets the DNA primase gene of *Fibralongavirus*.

When testing lytic activity using high phage titers 10^8^–10^10^ PFU mL^−1^ inhibition of growth was observed, which could mean the presence of tail-associated hydrolases or an abortive infection system [62], although no further experiments were conducted to analyse the mechanism of non-productive phage infection. This phenomenon was not caused by bacteriocin production, as confirmed by testing the antimicrobial activity of the supernatant from the bacterial culture and filtered phage lysate.

There are different approaches to describing the structure of staphylococcal phage genomes [63]. One way to classify phages without comparing modules is by describing the physical ends of the genome. The structure of the DNA packaging and head morphogenesis module was used to discriminate *cos* (Sfi21-like phages) from *pac* (Sfi11-like phages) phages [64]. The presence of a gene for homing endonuclease close to the gene for the small terminase is important for *cos* phages [65]. Phages QT1 and 2638A both support these characteristics for *cos* phages. It is similarly true for *Staphylococcus aureus* phage 77, which is their closest neighbour according to the phylogeny of Mcp and TerL (Figure 5). Siphophages mediate generalized transduction even to phage-resistant strains, [18] however we have not observed any bacterial sequences in phage sequencing reads in agreement with previous studies showing that *cos* phages transduce at low frequencies [21].

There is a major difference between phages QT1 and 2638A in the integration module (Figure 4). Although the domain structure of the integrases is identical (AP2 binding, SAM-like and catalytic domains), the amino acid sequence is dissimilar, and prophages integrate into different loci in the genome. A prophage that contains an identical integrase to phage QT1 is integrated into tRNA^Ser^ between the genes encoding for the ComK and SdrD proteins in the genome of *S. pseudintermedius* SL/154 [66]. In contrast, an integrase identical to that of phage 2638A is inserted into the gene for tRNA^Arg^ between genes encoding for the ClpP and WhiA proteins in the genome of *S. pseudintermedius* 104N (GenBank assembly accession number GCF_002847235).

The replication module is similar for both phages. The single-strand binding protein QBJ05148 was originally predicted to be a DUF2815-containing protein, but after tertiary structural prediction the model fitted the solved structure of an *Enterobacter* phage Enc34 single-strand binding protein [67] (PDB accession 5ODK). The domain of unknown function DUF2815 might become obsolete. There are minor differences between QT1 and 2638A in their DNA metabolism modules, especially in the presence of two predicted methyltransferases in the genome of phage QT1. The existence of methylases in staphylococcal phage genomes was observed at phages 80 or φ42 [68,69], however methylases are not widely distributed among staphylococcal phages. The role of methyltransferases in the phage genome is unknown. It is expected that methyltransferases protect the phage DNA from host restriction [70] or when the phage undergo lysogenic cycle, the lysogenic bacteria might become resistant to the infection by next phages [68]. Experiments with methylation-sensitive restriction endonucleases showed that DNA was cleaved. The reason might be that either different sites are methylated, the methyltransferases are dysfunctional, or methyltransferases process a different substrate than genomic DNA

Structural proteins were confirmed by both SDS-PAGE analysis and mass spectrometry (Figure 3; Table S2). The major capsid protein of QT1 is similar to the HK97 family of major capsid proteins but has a shorter Δ-domain consisting of approximately 60 residues. This domain is proteolytically cleaved by the adjacent protease and is not present in the mature virion. A hypothetical protein (GenBank accession QBJ05123) was detected via LC-MS/MS analysis. The position of this gene close to the tail length tape-measure protein (Tmp) was expected to act as a chaperone for Tmp during the assembly of the tail [71], but it seems that this protein might be somehow included in the structure of the tail itself.

The gene for the tail length tape-measure protein is the longest gene in the genome and Tmp is expected to work as a ruler to determine the length of the phage tail. With uniform coverage from the mass spectrometer, all domains are present in the virus particle. Thus, domains predicted to be a lysozyme-like domain and M23 peptidase domain may degrade peptidoglycan in a similar way as in phage vB_SauS-φIPLA35 [72] and could be a possible candidate protein for enzybiotics.

The central long tail fibre is distinctive for *Fibralongavirus* morphology (Figure 1). The full length of the tail fibre in the mature virion is supported by SDS-PAGE, where the corresponding band is visible in the untruncated length (162 kDa) corresponding to 1419 residues (Figure 3). The tail fibre from phage 2638A is 265 residues shorter (132 kDa) than the tail fibre from QT1, which manifests as a 17 nm shorter fibre under electron microscopy. With internal chaperon domain, there is probably no need for next tail fibre assembly protein as is the case with many other phages [73]. This long tail fibre might play a role in fast adsorption that was observed even on a phage resistant strain, or its length is required to attach to a membrane receptor under thick *S. pseudintermedius* cell wall [74]. Long tail fibres were described in *Bacillus* viruses γ and Wβ, *Escherichia* virus T5, *Salmonella* virus χ, and similar phages. Genetic analysis of *Bacillus* viruses γ and Wβ observed the tail fibre gene to be a mutation hotspot for host tropism and tail fibre mutations leading to larger plaque size [75]. In *Salmonella* virus χ, the long tail fibre serves for reversible adsorption to the flagellum of bacteria [76]. *Staphylococcus* lacks flagella, but this tail fibre might serve as an initial reversible binding receptor. After initial adsorption, a second unidentified receptor at the base of the tail might irreversibly bind to the host cell and mediate DNA ejection. The C terminal end of the tail fibre protein contains an intramolecular chaperone auto-processing domain. This domain mediates auto-proteolysis when is in trimeric form [55]. This might suggest another mechanism of adsorption. A change in the conformation after the initial contact of the tail fibre with the target molecule might activate the auto-processing domain and cleave the tail fibre, releasing another binding site that would irreversibly bind to bacteria. This approach would not require the presence of another protein in the genome of the phage.

The holins of both phages are almost identical. A major work that described phage 2638A was predominantly focused on the endolysin from this phage, because of its dissimilarity to other *S. aureus* endolysins [77,78,79,80]. The high amino-acid identity (95.7%–97.1%) of endolysins of phages 2638A, QT1, and the φSP119-1 prophage [56] indicate that endolysins of *Fibralongavirus* have similar properties. The endolysin of 2638A has a secondary translational start [77] mediated by a leucine codon (TTG) at position 180, which is common in the *Fibralongavirus* genus. We assume that two variants of endolysin are produced. The secondary translational start of QT1 has an identical sequence 18 nt prior and after the secondary starting codon. One endolysin variant contains the M23 peptidase, amidase and SH3 binding domains, and the other one is composed only of the amidase and SH3 binding domains. Although the endolysin of phage 2638A comes from *S. pseudintermedius*, it was used to protect mice from methicillin-resistant *S. aureus* and performed exceptionally well, protecting up to 100% of infected mice [79]. Applied research of the endolysin from QT1 would be beneficial for the further improvement of enzybiotics.

Most of the work on the classification of staphylococcal siphoviruses focuses on *S. aureus*, and the only non-S. *aureus* phage genus in ICTV taxonomy is *Sextaecvirus* of *Staphylococcus epidermidis.* However, further work is needed on the taxonomical position of non-S. *aureus* phages such as vB_SepiS-φIPLA5, vB_SepiS-φIPLA7, CNPH82 and PH15 that infect *S. epidermidis* [81,82] and are visible as a separate cluster (Figure 6). The description of the genus *Fibralongavirus* increases the number of known non-*S. aureus* genera and expands the current knowledge about the diversity of medically important non-S. *aureus* phages. The next member of genus *Fibralongavirus* could be prophage φSP119-1 (GenBank accession MK075004), if it can be induced. Along with the genus *Fibralongavirus*, there are at least two other clusters in *S. pseudintermedius* phages available in GenBank (Figure S4) and more research should be focused on those genera.

Considering the clinical background and presence of virulence factors in the genome of propagating strain 625, a different, safer propagating strain would be needed for the application of phages in therapy. The use of phages that contain integrase for phage therapy is unfavourable [83], however the described phage QT1 is active against clinical strains of *S. pseudintermedius* and could be used until a polyvalent phage is described and can be used as a gene source for enzybiotics.

## Supplementary Materials

The following are available online at https://www.mdpi.com/1999-4915/11/12/1143/s1: Figure S1: Adsorption curves for phage QT1 on different *Staphylococcus pseudintermedius* strains; Figure S2: Cryo-EM micrograph of phage QT1; Figure S3: Pulsed-field gel electrophoresis image of separated concatemers of phage QT1 genome; Figure S4: Heatmap of phages and prophages of *Staphylococcus pseudintermedius*; Table S1: Host range of phages QT1 and 2638A, and strain isolation source; Table S2: Proteomic report of proteins detected in LC-MS/MS; Table S3: Virulence factors detected in the genome of *Staphylococcus pseudintermedius* strain 625.
