# Supplementary material for: New Genus Fibralongavirus in Siphoviridae Phages of Staphylococcus pseudintermedius
Source: Viruses. 2019 Dec 10;11(12):1143. doi: 10.3390/v11121143 (PMC6950010; doi:10.3390/v11121143)
Supplement: Supplementary file 1 [file viruses-11-01143-s001.pdf]

viruses

MDPI

Supplementary materials

New genus *Fibralongavirus* in Siphoviridae phages of *Staphylococcus pseudintermedius*

Michal Zeman [1], Pavol Bárdy [1], Veronika Vrbovská [1], Pavel Roudnický [2], Zbyněk Zdráhal [2,3], Vladislava Růžičková [1], Jiří Doškař [1] and Roman Pantůček [1,*]

[1] Department of Experimental Biology, Faculty of Science, Masaryk University, Kotlářská 2, 611 37 Brno, Czech Republic; michal.zeman91@gmail.com (M.Z.); bardy.pavol@mail.muni.cz (P.B.); veronika.vrbovska@gmail.com (V.V.); vladkar@sci.muni.cz (V.R.); doskar@sci.muni.cz (J.D.)
[2] Central European Institute of Technology, Masaryk University, Kamenice 5, 625 00 Brno, Czech Republic; p.roudnicky@mail.muni.cz (P.R.); zdrahal@sci.muni.cz (Z.Z.)
[3] National Centre for Biomolecular Research, Faculty of Science, Masaryk University, Kamenice 5, 625 00 Brno, Czech Republic
* Correspondence: pantucek@sci.muni.cz; Tel.: +420-549-49-6379 (R.P.)

This document contains supplementary materials:

Figure S1. Adsorption curves for phage QT1 on different *Staphylococcus pseudintermedius* strains.

Figure S2. Cryo-EM micrograph of phage QT1.

Figure S3. Pulsed-field gel electrophoresis image of separated concatemers of phage QT1 genome.

Figure S4. Heatmap of phages and prophages of *Staphylococcus pseudintermedius*.

Table S1. Host range of phages QT1 and 2638A, and strain isolation source.

Table S2. Proteomic report of proteins detected in LC-MS/MS.

Table S3. Virulence factors detected in the genome of *Staphylococcus pseudintermedius* strain 625.

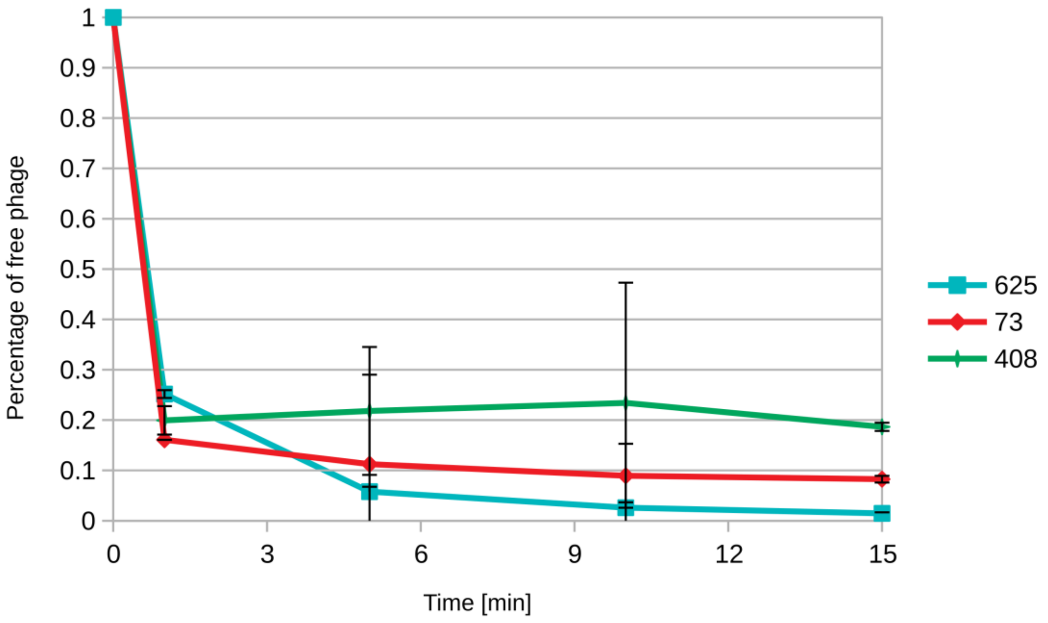

Figure S1. Adsorption curves for phage QT1 on different *Staphylococcus pseudintermedius* strains. Strain 625 is susceptible, strain 73 exhibited lysis from without, strain 408 was resistant to QT1. Points on the graph are mean values and the error bars correspond to standard deviations, based on three independent experiments.

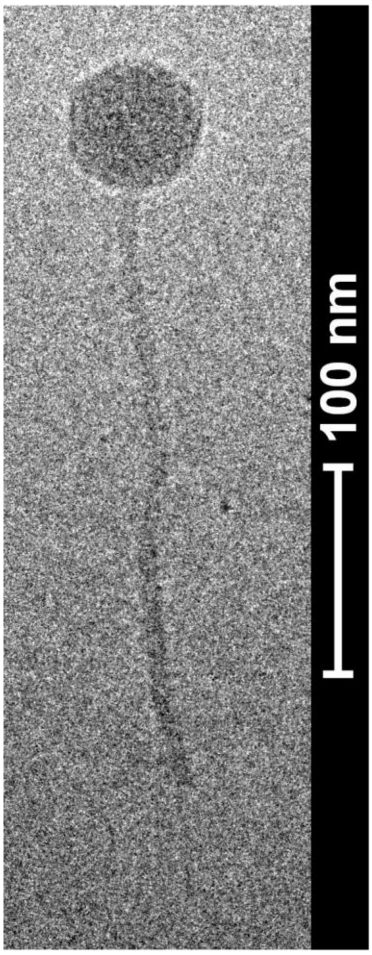

Figure S2. Cryo-EM micrograph of phage QT1. Tail fibre is barely visible on native phage.

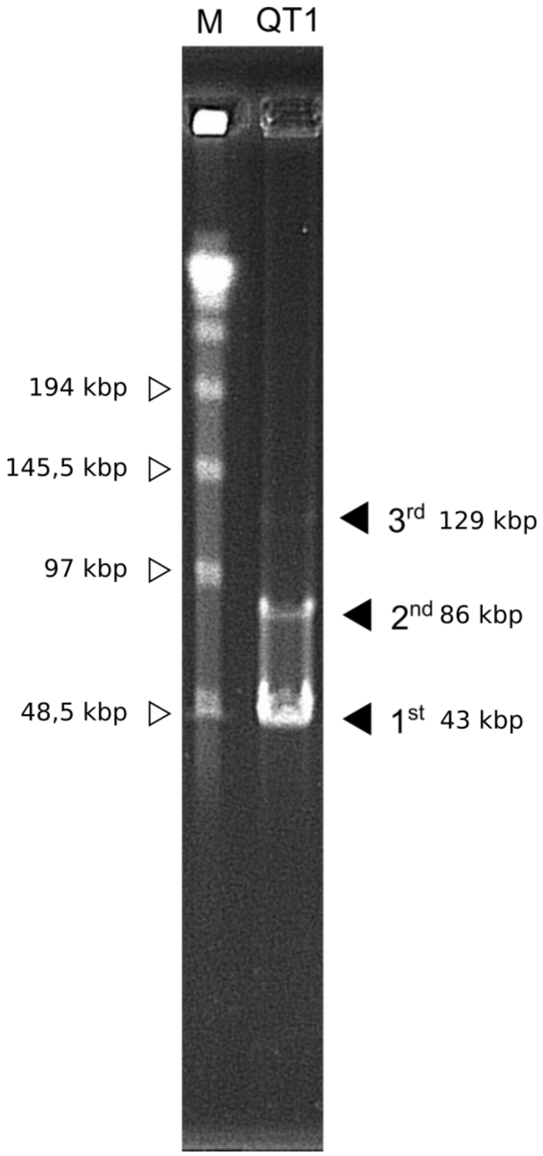

Figure S3. Pulsed-field gel electrophoresis image of separated concatemers of phage QT1 genome. At least trimers of the whole genome are visible. Lambda DNA concatemers were used as a marker (M).

	1	2	3	4	5	6	7	8	9	10	11	12	13	14	15	16	17	18	19
1. AP019560 *Staphylococcus* phage SP120	100	62	56	49	58	57	54	56	57	57	57	43	12	9	3	1	3	1	1
2. AP019562 *Staphylococcus* phage SP276	63	100	58	45	53	55	53	54	54	55	55	39	17	8	2	1	3	1	1
3. KX827369 *Staphylococcus* phage SpT152	55	56	100	52	55	60	58	60	59	59	59	40	12	7	1	2	3	3	1
4. MK075003 *Staphylococcus* phage phiSP44-1	46	42	50	100	47	53	52	55	53	53	53	45	9	4	1	2	1	1	0
5. AP019561 *Staphylococcus* phage SP197	57	52	55	49	100	71	74	73	71	70	71	39	9	5	3	3	3	2	1
6. KX827370 *Staphylococcus* phage SpT252	57	55	62	56	73	100	90	83	85	84	84	40	11	4	3	1	2	1	1
7. MF428479 *Staphylococcus* phage SN11	55	53	61	56	76	90	100	90	88	87	87	40	9	3	2	1	2	2	1
8. MF428478 *Staphylococcus* phage SN13	56	54	62	58	74	82	89	100	96	95	95	38	10	5	3	3	2	2	0
9. MF428480 *Staphylococcus* phage SN10	58	54	62	56	73	86	89	97	100	99	99	38	9	4	3	1	2	2	0
10. MF428481 *Staphylococcus* phage SN8	59	55	62	56	72	85	88	96	99	100	100	38	9	4	3	1	2	2	0
11. KX827368 *Staphylococcus* phage SpT5	59	55	61	56	73	85	88	96	99	100	100	38	9	4	3	1	2	2	0
12. MK075006 *Staphylococcus* phage phiSP119-3	39	35	37	43	37	36	37	35	35	34	34	100	11	4	6	1	10	6	1
13. MK075002 *Staphylococcus* phage phiSP38-1	11	15	11	9	8	11	9	9	8	8	8	12	100	41	11	6	11	7	4
14. KX827371 *Staphylococcus* phage SpT99F3	10	7	8	4	5	4	3	5	4	4	4	4	44	100	11	17	20	15	1
15. NC_007051 *Staphylococcus* phage 2638A	2	2	1	1	3	2	2	3	3	3	3	6	11	10	100	60	55	14	1
16. MK450538 *Staphylococcus* phage vB_SpsS_QT1	1	1	2	2	3	1	1	3	1	1	1	1	6	16	58	100	62	14	1
17. MK075004 *Staphylococcus* phage phiSP119-1	3	3	3	1	3	2	2	2	2	2	2	[illegible]	11	18	51	60	100	13	0
18. MK075001 *Staphylococcus* phage phiSP15-1	1	1	3	1	2	1	2	2	2	2	2	[illegible]	7	14	13	13	13	100	1
19. MK075005 *Staphylococcus* phage phiSP119-2	1	1	1	0	1	1	1	0	0	0	0	1	4	1	1	1	1	1	100

Fibralongavirus

Figure S4. Heatmap of phages and prophages of *Staphylococcus pseudintermedius* with cluster around phage QT1 based on fragmented nucleotide sequence alignment. The proposed genus *Fibralongavirus* is highlighted in text box. Phage names and GenBank accession numbers of used phages are labelled as in NCBI.

Table S1. Host range of phages QT1 and 2638A, and strain isolation source.

Strain	Sensitivity to QT1	Sensitivity to 2638A	Source	Species	Reference or culture collection
625#	+	+	Dog	*S. pseudintermedius*	Melter *et al.*, 2017
2854 (=HER 1283)	+	+	not available	*S. pseudintermedius*	HER, Slopek and Krzywy, 1985
CCM 2885	(+)	-	Healthy fox	*S. pseudintermedius*	CCM
CCM 4539	-	-	Human throat	*S. pseudintermedius*	CCM
CCM 4710	-	-	Human wound	*S. pseudintermedius*	CCM
CCM 7315T	(+)	-	Cat lung	*S. pseudintermedius*	CCM
CCM 7532	-	-	Dog dermatitis	*S. pseudintermedius*	CCM
CCM 7829	(+)	-	Dog adentitis	*S. pseudintermedius*	CCM
CCM 7830	(+)	-	Dog skin	*S. pseudintermedius*	CCM
CCM 7843	-	-	Human conjuctiva	*S. intermedius*	CCM
CCM 5739T	-	-	Pigeon	*S. intermedius*	CCM
29	-	-	Dog	*S. pseudintermedius*	Melter *et al.*, 2017
30	-	-	Dog	*S. pseudintermedius*	Melter *et al.*, 2017
33	(+)	-	Dog	*S. pseudintermedius*	Melter *et al.*, 2017
35	+	-	Dog	*S. pseudintermedius*	Melter *et al.*, 2017
73	-	-	Dog	*S. pseudintermedius*	Melter *et al.*, 2017
86	-	-	Dog	*S. pseudintermedius*	Melter *et al.*, 2017
105	-	-	Dog	*S. pseudintermedius*	Melter *et al.*, 2017
153	-	-	Dog	*S. pseudintermedius*	Melter *et al.*, 2017
156	-	-	Dog	*S. pseudintermedius*	Melter *et al.*, 2017
193	-	-	Dog	*S. pseudintermedius*	Melter *et al.*, 2017
236	-	-	Dog	*S. pseudintermedius*	Melter *et al.*, 2017
239	-	-	Dog	*S. pseudintermedius*	Melter *et al.*, 2017
252	-	-	Dog	*S. pseudintermedius*	Melter *et al.*, 2017
259	+	-	Dog	*S. pseudintermedius*	Melter *et al.*, 2017
279	-	-	Dog	*S. pseudintermedius*	Melter *et al.*, 2017
293	-	-	Dog	*S. pseudintermedius*	Melter *et al.*, 2017
297	-	-	Dog	*S. pseudintermedius*	Melter *et al.*, 2017
313	-	-	Dog	*S. pseudintermedius*	Melter *et al.*, 2017
383	-	-	Dog	*S. pseudintermedius*	Melter *et al.*, 2017
408	-	-	Dog	*S. pseudintermedius*	Melter *et al.*, 2017
497	-	-	Dog	*S. pseudintermedius*	Melter *et al.*, 2017
550	-	-	Dog	*S. pseudintermedius*	Melter *et al.*, 2017
552	-	-	Dog	*S. pseudintermedius*	Melter *et al.*, 2017
558	-	-	Dog	*S. pseudintermedius*	Melter *et al.*, 2017
571	-	-	Dog	*S. pseudintermedius*	Melter *et al.*, 2017
612	-	-	Dog	*S. pseudintermedius*	Melter *et al.*, 2017
621	(+)	-	Dog	*S. pseudintermedius*	Melter *et al.*, 2017
630	-	-	Dog	*S. pseudintermedius*	Melter *et al.*, 2017
666	-	-	Dog	*S. pseudintermedius*	Melter *et al.*, 2017
679	-	-	Dog	*S. pseudintermedius*	Melter *et al.*, 2017
748	-	-	Dog	*S. pseudintermedius*	Melter *et al.*, 2017
778	-	-	Dog	*S. pseudintermedius*	Melter *et al.*, 2017
802	-	-	Dog	*S. pseudintermedius*	Melter *et al.*, 2017
00/470	-	(+)	Human	*S. pseudintermedius*	Mališová *et al.*, 2019
02/172	-	-	Human	*S. pseudintermedius*	Mališová *et al.*, 2019
02/179	-	-	Human	*S. pseudintermedius*	Mališová *et al.*, 2019
02/264	-	(+)	Dog	*S. pseudintermedius*	Mališová *et al.*, 2019
02/438	-	-	Human	*S. pseudintermedius*	Mališová *et al.*, 2019
02/589	-	-	Human	*S. pseudintermedius*	Mališová *et al.*, 2019
02/682	-	(+)	Human	*S. pseudintermedius*	Mališová *et al.*, 2019
02/718	-	-	Human	*S. pseudintermedius*	Mališová *et al.*, 2019
14/858*	-	-	Tomcat wound	*S. pseudintermedius*	NRL/St
15/646*	-	-	Human skin abscess	*S. pseudintermedius*	NRL/St

+ sensitive at routine test dilution (RTD, 10^8 PFU mL^{-1}); (+) sensitive at RTD × 100, presence of plaques was considered as positive result; - resistant; # propagation strain for phage QT1; * *mecA* positive isolate
CCM – Czech Collection of Microorganisms, Masaryk University, Faculty of Science, Czech Republic
NRL/St – National Reference Laboratory for Staphylococci, National Institute of Public Health, Czech Republic
HER – Félix d'Hérelle Reference Center for Bacterial Viruses, Université Laval, Canada

Table S2. Proteomic report of proteins detected in LC-MS/MS. The proteome of phage QT1 was used as a reference database.

GenBank Accession	Description	MW [kDa]	# AAs	SAF	NSAF (%)	Sum (Coverage)	Sum (# Unique Peptides)	Sum(# PSMs)
QBJ05121.1	major tail protein with YjdB Ig-like domain [vB_SpsS_QT1]	32.8	301	1.16	25.23	0.82	14	348
QBJ05114.1	portal protein [vB_SpsS_QT1]	44.6	385	0.73	15.93	0.85	34	281
QBJ05117.1	head-tail adapter protein [vB_SpsS_QT1]	11.2	96	0.59	12.96	0.85	7	57
QBJ05116.1	major capsid protein [vB_SpsS_QT1]	43.2	395	0.58	12.59	0.81	33	228
QBJ05126.1	tail component [vB_SpsS_QT1]	56.1	494	0.25	5.52	0.72	32	125
QBJ05120.1	tail completion protein [vB_SpsS_QT1]	16.1	133	0.21	4.59	0.72	9	28
QBJ05118.1	head-closure protein [vB_SpsS_QT1]	14.2	120	0.21	4.55	0.78	7	25
QBJ05127.1	structural protein [vB_SpsS_QT1]	161.5	1419	0.20	4.31	0.64	78	280
QBJ05125.1	tail length tape-measure protein [vB_SpsS_QT1]	222.1	2031	0.14	2.98	0.56	101	277
QBJ05165.1	hypothetical protein [vB_SpsS_QT1]	6.9	58	0.07	1.50	0.36	2	4
QBJ05143.1	hypothetical protein [vB_SpsS_QT1]	9.6	80	0.06	1.36	0.36	3	5
QBJ05164.1	hypothetical protein [vB_SpsS_QT1]	11.7	100	0.06	1.31	0.52	4	6
QBJ05160.1	putative methyltransferase [vB_SpsS_QT1]	18.2	154	0.05	1.13	0.32	6	8
QBJ05123.1	hypothetical protein [vB_SpsS_QT1]	13.7	119	0.04	0.92	0.45	5	5
QBJ05113.1	terminase large subunit [vB_SpsS_QT1]	64.3	553	0.04	0.87	0.34	16	22
QBJ05115.1	Clp protease [vB_SpsS_QT1]	30.4	274	0.04	0.80	0.37	7	10
QBJ05139.1	immunoprotective extracellular protein [vB_SpsS_QT1]	24.3	215	0.03	0.71	0.62	7	7
QBJ05119.1	neck protein [vB_SpsS_QT1]	15.7	133	0.03	0.66	0.24	3	4
QBJ05153.1	S-adenosyl-L-methionine-dependent methyltransferase [vB_SpsS_QT1]	26.6	225	0.02	0.48	0.14	3	5
QBJ05148.1	nucleic acid-binding protein [vB_SpsS_QT1]	19.8	184	0.02	0.36	0.21	3	3
QBJ05132.1	N-acetylmuramoyl-L-alanine amidase [vB_SpsS_QT1]	55.3	486	0.01	0.31	0.16	6	7
QBJ05149.1	DNA polymerase A with ribonuclease H-like domain [vB_SpsS_QT1]	73.8	653	0.01	0.30	0.16	9	9
QBJ05147.1	putative DNA replication ATP dependent nuclease [vB_SpsS_QT1]	44.2	388	0.01	0.28	0.12	4	5
QBJ05166.1	DNA primase [vB_SpsS_QT1]	94.2	816	0.01	0.21	0.11	7	8
QBJ05169.1	DNA helicase [vB_SpsS_QT1]	52.6	452	0.01	0.14	0.08	3	3

MW – calculated molecular weight; SAF – Spectral Abundance Factor; NSAF – Normalized Spectral Abundance Factor; PSM - peptide spectrum match

Table S3. Virulence factors detected in the genome of *Staphylococcus pseudintermedius* strain 625.

Product	Abbreviation	Locus tag	Coverage	E value	Identity	Predicted function	Best hit*
**Major factors**							
Leukocidin S-I, gamma-hemolysin	LukS-I	GB868_01705	100	0	100	pore forming	WP_014613568
Leukocidin F-I, gamma-hemolysin	LukF-I	GB868_01700	100	0	99.7	pore forming	WP_014613567
Phospholipase C, beta-hemolysin	Hlb	GB868_04675	90	0	74.58	pore forming	P09978
Delta-hemolysin	Hld	GB868_08280	100	2E-7	65.38	pore forming	P0A0M1
Hemolysin *S. pseudintermedius*	HlSp	GB868_01530	93	4E-7	42.86	pore forming	P85219
Alpha-class phenol soluble modulin	PSMα	GB868_09320#	100	2E-17	95.65	pore forming	WP_103214038
Beta-class phenol soluble modulin	PSMβ	GB868_09315	100	6E-23	100	pore forming	WP_014614190
Enterotoxin C2 – bovine	SECb	GB868_07230	100	3E-122	57.6	superantigen	P34071
**Enzymatic function**							
Exfoliative toxin B	Etb	GB868_07225	98	7E-112	56.3	skin scalding	P09332
ATP-dependent Clp protease proteolytic subunit ClpP	ClpP	GB868_07115	99	2E-145	94.85	protease	Q5HQW0
ATP-dependent Clp protease proteolytic subunit clpB	ClpB	GB868_05635	99	0	83.37	chaperone heatshock	Q6GIB2
ATP-dependent Clp protease proteolytic subunit clpC	ClpC	GB868_04880	100	0	90.48	chaperone heatshock	Q5HRM8
ATP-dependent Clp protease proteolytic subunit clpX	ClpX	GB868_02335	100	0	90.24	protease subunit	A5ITJ9
Aureolysin	Aur	GB868_05855	100	0	62.4	immune evasion	P81177
Staphylocoagulase precursor	Coa	GB868_10500	49	5E-19	30.01	blood clotting	P07767
**Biofilm**							
Intercellular adhesin protein A	IcaA	GB868_03145	99	0	61.61	biofilm production	Q6G608
Intercellular adhesin protein B	IcaB	GB868_03155	84	5E-113	56.73	biofilm production	Q5HCM9
Intercellular adhesin protein C	IcaC	GB868_03160	98	2E-131	55.76	biofilm production	P69518
Intercellular adhesin protein D	IcaD	GB868_03150	88	9E-14	33.33	biofilm production	P69519
Veg protein	Veg	GB868_10350	89	4E-37	63.29	biofilm regulation	P37466
**Quorum sensing**							
Accessory gene regulator protein A	AgrA	GB868_08300	100	1E-160	83.61	quorum sensing	P0A0I5
Accessory gene regulator protein B	AgrB	GB868_08285	100	1E-122	89.36	quorum sensing	P61649
Accessory gene regulator protein C	AgrC	GB868_08295	99	0	93.49	quorum sensing	WP_096636795
Accessory gene regulator protein D	AgrD	GB868_08290	100	8E-24	95.56	quorum sensing	WP_063278585
**Immune evasion, msscram**							
Super-antigen protein	Ssl11	GB868_10495	100	4E-54	44.26	superantigen	WP_031788342
Secretory antigen ssaA protein	SsaA1	GB868_08840	100	2E-76	56.8	immune evasion	Q5HLV2
Secretory antigen ssaA protein	SsaA2	GB868_08860	89	3E-41	63.39	immune evasion	Q2G2J2
Immunoglobulin G-binding protein SBI	Sbi	GB868_03995	68	3E-51	48.22	immune evasion	A7X659
Major histocompatibility complex II analog	Map	GB868_02100	98	9E-42	42.29	immune evasion	P69775
Fibronectin binding protein A	SpsL	GB868_03575	100	0	89.4	mscramm	ADX75396
Fibronectin-binding protein and Serine-aspartate repeat-containing protein	SpsD	GB868_00855	90	0	87.9	mscramm	ADX76659
Fibronectin/fibrinogen and RNA binding protein	Frbp	GB868_10105	99	0	47.1	mscramm	O34693
Serine-aspartate repeat-containing protein E	SdrD	GB868_05385	88	2E-149	36.46	mscramm	Q6GBS4
Adhesion lipoprotein	EfaA	GB868_04210	97	1E-98	47.52	mscramm	NP_815739
Cell surface elastin binding protein	EpbS	GB868_00450	99	8E-44	33.66	mscramm	NP_646186
**Bacteriocins**							
Microcin C7 self-immunity protein MccF	MccF	GB868_01200	96	5E-34	29.25	bacteriocin resistance	Q47511
Colicin V production protein	CvpA	GB868_09450	100	0	100	bacteriocin	WP_037543270
Four-helix bundle bacteriocin BacSp222	BacSp222	GB868_11725	100	2E-31	100	bacteriocin / immuno-modulation	A0A0P0C3P7

*swiss-prot as a reference database, otherwise well documented protein; mscramm - microbial surface components recognizing adhesive matrix molecules; # correct coordinates are contig WJOJ01000014 - nucleotide 16837..16908
